# Paper-based detection of HIV-1 drug resistance using isothermal amplification and an oligonucleotide ligation assay

**DOI:** 10.1016/j.ab.2017.12.008

**Published:** 2018-03-01

**Authors:** Mary E. Natoli, Brittany A. Rohrman, Carolina De Santiago, Gert U. van Zyl, Rebecca R. Richards-Kortum

**Affiliations:** a Department of Bioengineering, Rice University, 6100 Main St MS-142, Houston, TX 77005, USA; b Omniome, Inc., 10575 Roselle St, San Diego, CA 92121, USA; c National Health Laboratory Service, Tygerberg Business Unit, Coastal Branch, South Africa; d Division of Medical Virology, Stellenbosch University, Parow, South Africa

**Keywords:** Recombinase polymerase amplification, HIV drug resistance, Lateral flow, Oligonucleotide ligation assay

## Abstract

Regular HIV-1 viral load monitoring is the standard of care to assess antiretroviral therapy effectiveness in resource-rich settings. Persistently elevated viral loads indicate virologic failure (VF), which warrants HIV drug resistance testing (HIVDRT) to allow individualized regimen switches. However, in settings lacking access to HIVDRT, clinical decisions are often made based on symptoms, leading to unnecessary therapy switches and increased costs of care. This work presents a proof-of-concept assay to detect M184V, the most common drug resistance mutation after first-line antiretroviral therapy failure, in a paper format. The first step isothermally amplifies a section of HIV-1 *reverse transcriptase* containing M184V using a recombinase polymerase amplification (RPA) assay. Then, an oligonucleotide ligation assay (OLA) is used to selectively label the mutant and wild type amplified sequences. Finally, a lateral flow enzyme-linked immunosorbent assay (ELISA) differentiates between OLA-labeled products with or without M184V. Our method shows 100% specificity and 100% sensitivity when tested with samples that contained 200 copies of mutant DNA and 800 copies of wild type DNA prior to amplification. When integrated with sample preparation, this method may detect HIV-1 drug resistance at a low cost and at a rural hospital laboratory.

## Graphical abstract

**Figure:**
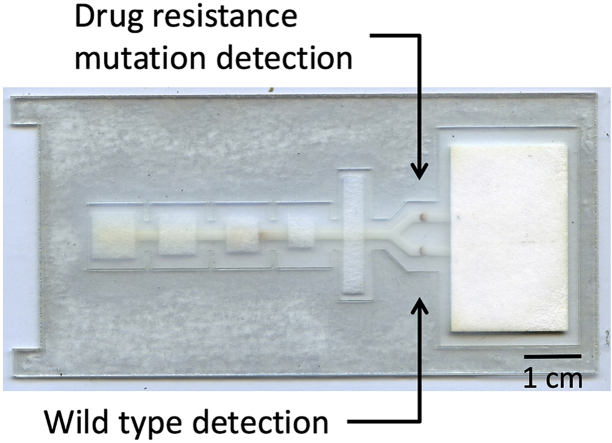

## Introduction

The number of deaths each year due to HIV has been decreasing, from a high of 2.3 million in 2006 to the current 1 million in 2016 [1,2]. Largely because of the introduction of lateral flow antibody tests, patients on antiretroviral therapy (ART) are now predicted to have near-normal life expectancy, and effective treatment of HIV to achieve viral load suppression is increasingly recognized as an important strategy for individual benefit and to prevent new infections at the population level [3,4]. However, the World Health Organization (WHO) reports that the success of ART is threatened by the emerging problem of acquired and transmitted drug resistance [5]. In some areas, including East Africa, resistance rates to non-nucleoside reverse transcriptase inhibitors (NRTIs) are above 10% [5]. Furthermore, between 10% and 30% of people receiving a first-line ART regimen will develop virological failure (VF) (viral load >1000 copies/mL) at some point during their treatment [[6], [7], [8]].

In high-resource settings, patients undergo phenotypic or genotypic HIV drug resistance testing at the time of diagnosis or treatment initiation, and after any instance of VF. However, these methods cost a minimum of $200 per sample and require extensive infrastructure, making resistance testing infeasible for many of the same low-resource settings that experience the greatest burden of HIV. Where resistance testing is not available, current WHO guidelines recommend an immediate adherence intervention when VF is detected, followed by a repeated viral load test three months later [3]. If the second viral load test confirms VF, a switch to second-line ART is recommended. Despite this recommendation, in practice, HIV care providers often do not switch patients immediately, due to concerns that VF resulted from non-adherence. Currently, excluding non-adherence as a cause of high viral load is challenging; existing adherence measurement tools rely on self-reporting measures, which are inadequate [9].

Two thirds of the 36.7 million people living with HIV/AIDS worldwide reside in sub-Saharan Africa, where access to resistance testing and even viral load monitoring is limited [1,10]. Lack of infrastructure in such areas and the high cost of treatment monitoring have resulted in a reliance on clinical monitoring only, or clinical monitoring in combination with CD4 monitoring [3]. These measures do not identify treatment failure with high sensitivity or specificity, and clinical and immunologic monitoring of treatment can result in delayed or unnecessary therapy switches to more expensive second line drugs [13]. Point-of-care (POC) or near-point-of-care assays are likely to be the most useful option to detect key DRMs in the most remote settings because they have the potential to detect low levels of resistance and operate at a low cost [11,12]. An inexpensive POC device that discriminates between patients who require adherence interventions from those who require a therapy switch to a second-line regimen could increase access to crucial resistance testing.

Such a test requires five main steps: (1) sample preparation to isolate nucleic acids from a patient sample of whole blood, (2) conversion of viral RNA to cDNA, (3) amplification of cDNA to detectable levels, (4) discrimination of wild type and mutant viral sequences, which differ by only a single nucleotide, and (5) visual readout of the test results. A number of technologies have been recently implemented to amplify nucleic acids at a single temperature, thus eliminating the need for thermal cycling equipment. One such technology, recombinase polymerase amplification (RPA), offers significant advantages for both instrumentation and assay development and has been used recently in low-cost diagnostics for several infectious diseases, including HIV [[13], [14], [15], [16]]. For example, RPA tolerates impure samples, amplifies DNA to detectable levels in as few as 5 min, and uses RPA reagents that are available in a lyophilized form that can be transported to the POC without requiring cold chain storage [13,17]. In addition, RPA operates at a wide range of temperatures. By including a reverse transcriptase enzyme, RPA is also able to reverse transcribe and amplify RNA targets. Thus, RPA is a good candidate technology for the amplification step of a POC HIV drug resistance assay.

To discriminate amplified wild type and mutant DNA sequences, short oligonucleotide probes can be employed in an oligonucleotide ligation assay (OLA) [[18], [19], [20], [21], [22]]. In an OLA, a common oligonucleotide (oligo) probe complementary to the region adjacent to the mutation is ligated to a wild type- or mutant-specific probe, resulting in a dual-labeled oligo that can be detected in an enzyme-linked immunosorbent assay (ELISA). Because ligation only occurs when the probes and amplified sequence are perfectly matched, OLA accurately detects single nucleotide differences [19].

The final step of an HIV drug resistance detection assay is differential detection of wild type and mutant OLA products. Traditional ELISA has been used to detect and discriminate between differently-tagged OLA products [23]. Although this provides quantitative results, 96-well assays require infrastructure, expensive machinery, and personnel not available in many low-resource settings. Recently, ELISAs have been implemented in a paper format [24,25]. These tests can be performed rapidly with small volumes of sample and have a limit of detection approaching that of standard ELISA. A recent study implemented a paper-based readout of the OLA for detecting HIV drug resistance in 45 min [22]. Advances in lateral flow network design have allowed for sequential reagent delivery without the need for pipettes. Reagents are added to glass fiber pads, the device is folded, and capillary action delivers the reagents sequentially to the test region without further user input. Another study has shown the feasibility of pre-drying reagents in paper networks for up to three months [26]. Paper-based tests are inexpensive, disposable, and easy to operate and interpret. In addition, several materials have already been well characterized for lateral flow techniques. Therefore, a paper-based ELISA that implements lateral flow detection of OLA products would be appropriate for a rapid, low-cost HIV drug resistance test.

In this paper, we demonstrate proof-of-concept for a modular assay that includes RPA-based isothermal amplification of the region of HIV-1 DNA that may contain four single nucleotide mutations associated with drug resistance, ligation of tagged oligonucleotide probes complementary to the amplified sequence (OLA), and sensitive and specific detection of the ligated products via a paper-based lateral flow ELISA with visual readout. The assay currently detects the most common drug resistance mutation, M184V, and can be adapted to detect other high-impact mutations.

## Materials and methods

### Preparation of synthetic HIV-1 DNA

**Fig. 1:**
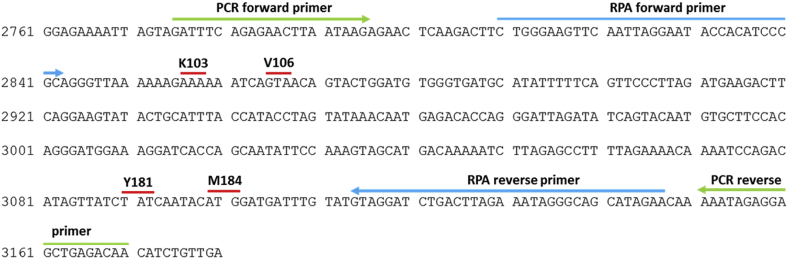
A representation of the DNA preparation and amplification scheme. The entire block is a 420-bp section of the HIV *pol* gene (nt 2761 to 3180) that was purchased from Integrated DNA Technologies. The green arrows indicate the sections targeted by PCR primers for DNA standard preparation. The blue arrows indicate the sections targeted by RPA primers for the integrated assay. The RPA assay amplifies a region that contains several major drug resistance mutations, K103N, V106M, Y181C, and M184V (wild type codons denoted with red lines).

Synthetic gBlock stocks (Integrated DNA Technologies) of a 420-bp region (HXB2 reference nucleotide positions: 2761 to 3180) of the *pol* gene were used to obtain template DNA for the assay. Two separate stocks were used, one encoding the sequence for wild type HIV-1 and other encoding the sequence that includes the M184V drug resistance mutation. A 396-bp section of each gBlock was amplified by PCR to obtain additional stock DNA and determine concentration standards (Fig. 1). 55.4 ng of gBlock DNA was added to 50 μL reaction mixtures containing 250 μM of forward (5′- CTAAAGTCTCTTGAATTATTC-3′) and reverse primers (5′-TTGTCTCAGCTCCTCTATTT-3′), 100 μM dNTPs, 5× Phusion buffer HF, and 1 U of Phusion HF DNA Polymerase (New England Biolabs). Cycling conditions consisted of a denaturation step of 60 s at 98 °C, 40 cycles of 15 s at 98 °C, 30 s at 48 °C, and 30 s at 72 °C, followed by an extension step of 5 min at 72 °C. Each reaction was purified using a QIAquick PCR Purification kit (Qiagen) using the microcentrifuge instructions and eluted in 50 μL of Millipore water. PCR products were visualized by electrophoresis on 2% agarose gels in TAE buffer, and gel extracted using a QIAquick Gel Extraction kit (Qiagen) with the microcentrifuge instructions. The concentration of the purified DNA was determined by measuring the OD280 with the NanoDrop, and the DNA copy number was calculated. Serial dilutions were made in T[0.1]E buffer (10 mM Tris pH 8, 0.1 mM EDTA) to obtain working concentrations in the range of 1-10^6^ copies per 10 μL and were stored at 4 °C in Lo-Bind Eppendorf tubes. For experiments studying mixtures of wild type and mutant DNA, these wild type and mutant stocks were combined in a 4:1 ratio for each concentration, and the same stock solution was used for all experiments.

### Amplification by recombinase polymerase amplification (RPA)

#### RPA assay design

We chose to amplify a sequence that contains several major drug resistance mutations so that the resulting amplicons could be used for OLA detection of more than one mutation. We designed and screened eight sets of potential forward and reverse primers (Table S-1) and selected the pair that had the best amplification efficiency. The selected primers amplify a 338-base-pair region (nt 2810 to 3147) of the *pol* gene that contains the resistance mutations M184V, K103N, Y181C, and V106M (Fig. 1).

#### RPA methods

RPA reactions were assembled according to the manufacturer's instructions (TwistAmp Basic kit, TwistDx, United Kingdom). Each 50 μL reaction contained 29.5 μL rehydration buffer, 2.4 μL forward primer (5′-GACCCTTCAAGTTAATCCTTATGGTGTAGGGCG-3′) [10 μM], 2.4 μL reverse primer (5′-TTCTATGCTGCCCTATTTCTAAGTCAGATCCTAC-3′) [10 μM], 3.2 μL water, one dehydrated enzyme pellet, 2.5 μL magnesium acetate, and 10 μL of either HIV-1 DNA template or deionized water for negative controls. All primer oligonucleotides were purchased from Integrated DNA Technologies (Novato, USA). RPA reactions were incubated in a heat block at 37**°**C for 45 min. Following amplification by RPA, each reaction was purified using a QIAquick PCR Purification kit (Qiagen) using the microcentrifuge instructions and eluted in 50 μL of Millipore water for visualization by gel electrophoresis, or 30 μL of Millipore water for increased concentration if continuing the assay. 50 μL elutions were run on a 2% agarose gel with ethidium bromide staining to check for successful amplification and the size of the amplicons.

### Drug resistance discrimination by oligonucleotide ligation assay (OLA)

#### OLA assay design

**Fig. 2:**
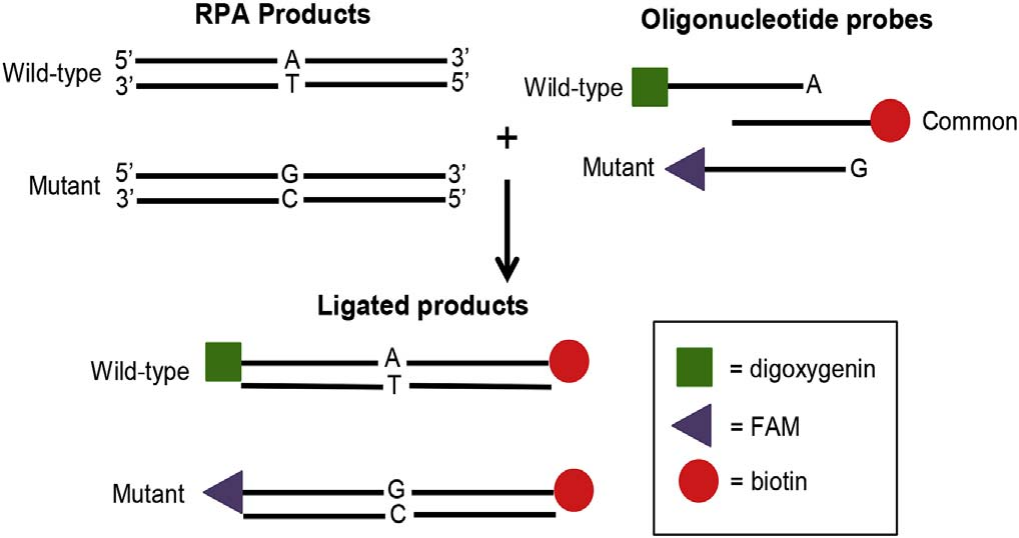
Oligonucleotide ligation assay. Schematic of oligonucleotide probe ligation over a site of interest.

To discriminate between amplified wild type and mutant sequences, we implemented an oligonucleotide ligation assay (OLA). Fig. 2 shows a schematic of the OLA; two reporter probes, specific to either the wild type or mutant sequence and labeled at the 5′ end with reporter molecules (digoxigenin or fluorescein amidite [6-FAM]), are annealed to the target sequence. The reporter probes differ only by a single nucleotide at the 3′ end, which is the site of the drug resistance mutation. A 5′-phosphorylated and 3′-biotinylated common probe anneals to the target sequence at the next nucleobase. Ligation of the two probes occurs when there is perfectly complementary base pairing between the 3′ end of the reporter probe and the target DNA sequence. The result is a single oligonucleotide having a biotin group at the 3′ end and either a digoxigenin or FAM group at the 5′ end. Ligation does not occur if the reporter probe does not match the target sequence [18].

##### OLA methods

Oligonucleotides used in the ligation assay were chosen based on previous work [19]. The sequences are: WT (5′-dig-AGACATAGTTATCTATCAATACA-3′), mutant (5′-f-AGACATAGTTATCTATCAATACG-3′), and common (5′-p-TGGATGATTTGTATGTAGGATC-bio-3′) (dig = digoxigenin, f = 6-FAM [fluorescein amidite], p = phosphate, bio = biotin). For the ligation assay, 2 μL of RPA-amplified product, 3 μL of 10× Taq DNA ligase buffer (New England Biolabs [200 mM Tris-HCl, 250 mM potassium acetate, 100 mM magnesium acetate, 10 mM NAD 1, 100 mM DTT, 1% Triton X-100, pH 7.6]), 3 μL of each oligonucleotide probe [10 μM], 1.2 μL of Taq DNA ligase (New England Biolabs), and 14.8 μL of Milli-Q^®^ water were added to each reaction for a total reaction volume of 30 μL. 40 cycles of 93**°**C for 30 s and 33**°**C for 30 s were performed. Following the OLA, each reaction was purified using a Qiaquick Nucleotide Removal Kit (Qiagen) in order to remove free nucleotides while retaining the ligated products. The ligation product was eluted in the final step using 30 μL of Milli-Q^®^ water. For use in the lateral flow portion of the assay, 20 μL of phosphate buffered saline (PBS) with 0.05% Tween 20^®^ (PBST) was added to the 30 μL of purified OLA product in order to promote fluid flow, and the sample was vortexed to mix.

Purified OLA products were visualized on a 10% denaturing PAGE gel with SYBR gold staining. Synthetic ligation products purchased from Integrated DNA Technologies (IDT) were run in adjacent lanes for a size comparison (WT: 5′-dig-AGACATAGTTATCTATCAATAC**A**TGGATGATTTGTATGTAGGATC-bio-3’; M184V: 5′-FAM- AGACATAGTTATCTATCAATAC**G**TGGATGATTTGTATGTAGGATC-bio-3′). In one experiment to verify the specificity of the OLA and ensure that the common probe would not ligate with a reporter probe that was not perfectly matched to the target DNA, only one reporter oligo was added to each reaction. The missing volume was replaced with an equivalent volume of water. For this experiment, OLA was performed as described above, but instead of including both mutant and wild type probe, the four samples included, respectively: WT target and WT probe, WT target and mutant probe, mutant target and WT probe, mutant target and mutant probe. If the assay is specific, a product should be produced when the target matches the included probe, and not when the target and included probe are mismatched. Products were visualized as above.

### Detection of labeled products by lateral flow assay (LFA)

#### Fabrication of LF strips

**Fig. 3:**
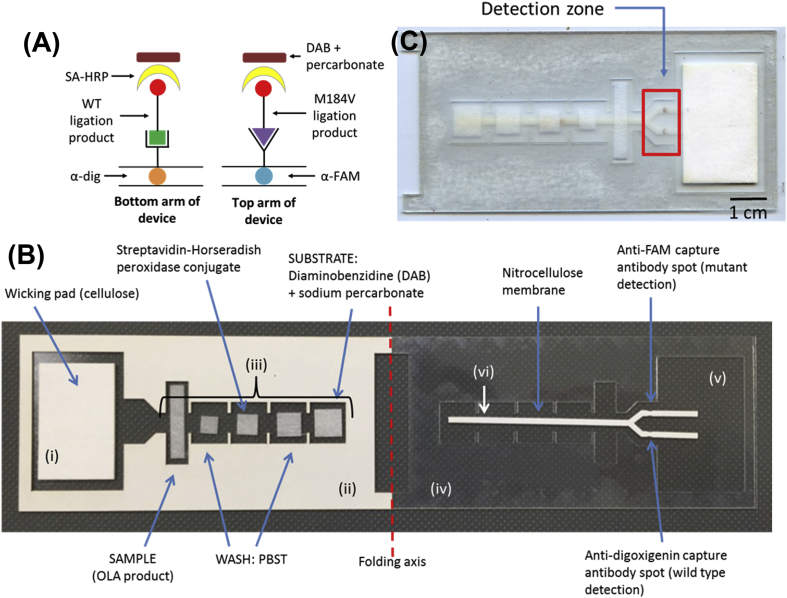
Lateral flow ELISA design. (A) A side view of the detection zone of the ELISA format for this lateral flow assay. OLA products are captured and detected by an enzyme-substrate mechanism. (B) Image of the lateral flow ELISA to discriminate HIV-1 mutant and wild type OLA products. After reagents are added, the adhesive backing on the left is removed and the device is folded and sealed along the folding axis to initiate flow and sequential delivery of reagents. The device parts indicated are: (i) cellulose wicking pad; (ii) adhesive acetate (paper side up); (iii) glass fiber pads; (iv) acetate; (v) adhesive acetate (paper backing removed and adhesive side up); (vi) nitrocellulose. (C) Image of the lateral flow design once folded. Starting with the OLA product contained in the glass fiber pad closest to the wicking pad, reagents flow sequentially across the detection zone.

All materials were cut using a 60-watt CO_2_ laser cutter (Universal Laser Systems). A nitrocellulose membrane (Millipore, Billerica, MA) was cut in a forked shape (Fig. 3). 0.2 μL each of goat polyclonal antibody to FITC (Abcam, ab19224) and a sheep antibody to digoxigenin (Abcam, ab64509) were spotted onto the detection region of the nitrocellulose membrane at a concentration of 1 mg/mL and 2 mg/mL respectively. Membranes were incubated at 37**°**C for 1 h, then soaked in a blocking solution (5% sucrose, 2% BSA, 0.25% PVP, 0.1% Tween 20^®^ in PBS, filtered through a 0.22 μm filter) on a shaker for 30 min at room temperature. The membranes were then incubated at 37**°**C for 90 min.

To construct the devices, a rectangular piece of adhesive acetate (Blick Art Materials) was used as a base for the device (Fig. 3B, v), and the paper was first removed to reveal the adhesive side. A template of the same material with cutouts for the other device components was adhered on the left, paper side up (Fig. 3B, ii), while an identical template made of regular acetate (Blick Art Materials) was adhered on the right (Fig. 3B, iv). With the templates in place, five glass fiber pads of varying sizes (Ahlstrom 8951, Helsinki, Finland) were placed onto the adhesive base using tweezers (Fig. 3B, iii). Glass fiber pad size was chosen according to the volume the pad would need to hold. A cellulose wicking pad (Millipore, Billerica, MA), designed to pull fluid towards it through the device, was also adhered to the base (Fig. 3B, i). The nitrocellulose membrane, once dry, was handled by the forked side only with tweezers and was adhered to the right side of the device with its paper side up (Fig. 3B, vi).

#### Assay design

To detect OLA products with minimal user input and at the POC, we adapted traditional ELISA detection to a paper LFA format, shown in Fig. 3A, based on the design original published by Yager et al. [24]. Based on the fluid wicking properties of paper, capillary action pulls reagents toward a wicking pad, allowing sequential flow of reagents and thus sequential binding to take place. First, anti-FAM and anti-digoxigenin antibodies are dried into nitrocellulose. When an OLA product flows over this spot, these antibodies selectively bind the reporter molecules FAM and digoxigenin on the 5′ end of each ligated product. Since the FAM moiety is attached to the mutant oligo probe but not to the wild type probe, the anti-FAM spot captures OLA products containing mutant DNA, while products that contain only wild type DNA continue to flow over it. The anti-digoxigenin spot captures OLA products containing wild type DNA. A forked design in the nitrocellulose paper allows the OLA product to split and flow separately over each spot, eliminating the possibility that aggregation will reduce sequential flow.

After flowing a wash step of PBST through the device, a streptavidin-horseradish peroxidase conjugate (SA-HRP-40) is flowed in order to detect the bound OLA product. Streptavidin-HRP will bind to the biotin moiety on the 3′ end of any OLA product that has bound to a detection spot. Following a second wash step of PBST, a mixture of the substrate 3,3′-diaminobenzidine (DAB) and sodium percarbonate flows over the complex. Hydrogen peroxide provided by sodium percarbonate reacts with the colorimetric substrate DAB, creating a visible brown coloration at the capture antibody spot.

#### Assay methods

To activate the assay, the following reagents were added to the glass fiber pads as depicted in Fig. 3B. From right to left, 30 μL of DAB solution (sodium percarbonate at a concentration of 0.5% w/v added fresh to a 0.5 mg/mL solution of 3,3′-diaminobenzidine [Sigma Aldrich, Saint Louis, MO] in water), 25 μL of PBST, 20 μL of streptavidin-HRP-40 conjugate solution ([Abcam, ab7403] diluted 1:1500 in PBST), and 15 μL of PBST were added. 50 μL of purified ligated product were added to the left-most rectangular glass fiber pad. The backing of the adhesive acetate on the left half of the device was removed to reveal the adhesive, and the device was folded and sealed. This brought the nitrocellulose membrane in contact with the glass fiber pads containing reagents, and induced reagent flow towards and over the detection zone (Fig. 3C). A clear signal was visible after 45 min, and strips were imaged once dry.

### Image capture and quantification

Assay membranes were imaged with a flatbed scanner (Epson) in 24-bit RGB mode at a resolution of 600 dpi. The signal-to-background ratio (SBR) of detection spots to background was calculated using a custom MATLAB script adapted from Grant et al. [25]. A fixed size square region of interest (ROI) was placed by the user over each capture region in an image. A rectangular ROI was placed over the portion of the strip between the capture region and wicking pad to obtain a background reading. To determine the signal, the complement of the image was first computed such that higher pixel intensity corresponded to a higher signal. The test signals were defined by calculating the mean of the maximum pixel intensity for each row of the test ROIs. The background signal was calculated identically using the background ROI. LFA results are expressed using the SBR.

### Integrated assay experimental design

To assess the specificity of the integrated assay, samples containing 10^5^ copies of wild type only DNA or M184V mutant only DNA were added as input to the RPA reaction and run through the integrated assay as described above. This was to ensure that no amplified wild type DNA would bind to the mutant detection spot, and no mutant DNA would bind to the wild type detection spot. Deionized water was used as a sample in a negative control RPA reaction to ensure that reagents associated with RPA and OLA would not result in false positive signals. Reactions were run in triplicate. Once the lateral flow strips were dry, the devices were scanned and analyzed as described above. A positive signal was defined objectively as a SBR greater than three standard deviations above the average negative control SBR. To determine the limit of detection of the assay when tested with 80% wild type and 20% mutant DNA, 10, 10^2^, 10^3^, 10^4^, or 10^5^ copies of the mixed DNA were added to the RPA reaction and the assay was run and analyzed in the same way. Because all three steps of the assay could contribute to its sensitivity, we found it best to consider the assay as a system and describe the input in number of copies in the pre-amplified sample, which would be closest to a clinical specimen. The output of the system is described as SBR. Reactions were run in triplicate.

## Results

### RPA assay performance

**Fig. 4:**
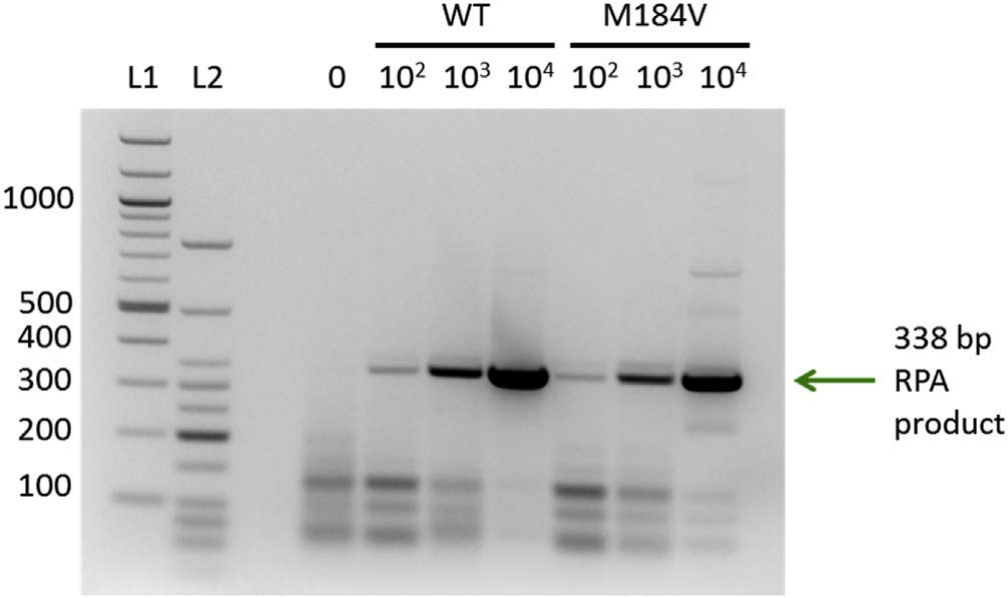
Purified RPA product from varying starting copy numbers run on a 2% agarose gel with ethidium bromide staining. L1 = 100 bp DNA ladder (New England Biolabs); L2 = Low Molecular Weight DNA ladder (New England Biolabs). Arrow indicates the 338-bp product; left side labels indicate fragment lengths in base pairs.

Gel electrophoresis of purified RPA products shows the amplification of a 338 bp sequence (Fig. 4), as expected using primers 4DR-F5 and 4DR-F7. Amplification is similar for both wild type and M184V HIV DNA, and a lower limit of 100 copies of target DNA is detectable via gel electrophoresis.

### OLA performance

**Fig. 5:**
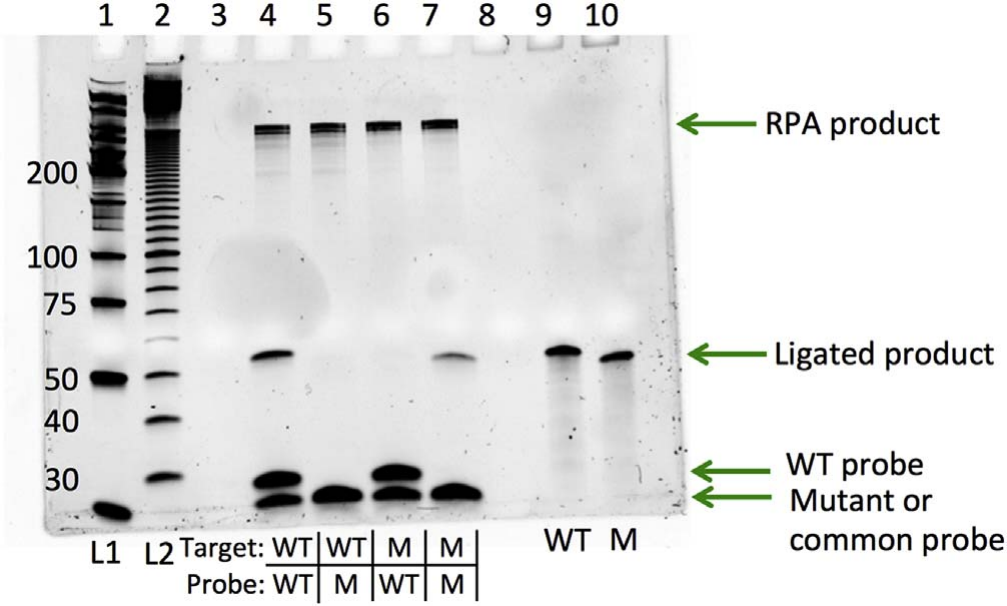
OLA products (lanes 4–7) and the synthetic expected sequences (lanes 9 and 10) run on a 10% denaturing PAGE gel with SYBR gold staining. L1 = Low Molecular Weight DNA Ladder (New England Biolabs); L2 = 10 bp DNA Ladder (New England Biolabs); left side labels indicate fragment lengths in base pairs. (For interpretation of the references to color in this figure legend, the reader is referred to the Web version of this article.)

Successful ligation of the common oligonucleotide probe (22 bp) to either the mutant-specific or the wild type-specific oligonucleotide (23 bp) should result in a 45-bp product. Fig. 5 shows the presence of such a product when matching target and probe sequences are present in the mixture, and the absence of such a product when the matching target and probe sequences are not both present.

The 45-bp ligated product in lanes 4 and 7 is similar in size to that of synthetic products (Integrated DNA Technologies) in lanes 9 and 10. Slight differences in migration between the mutant and wild type ligated products and probes despite their equal length are likely due to the reporter molecules FAM and digoxigenin differentially affecting migration behavior.

### Lateral flow assay specificity

**Fig. 6:**
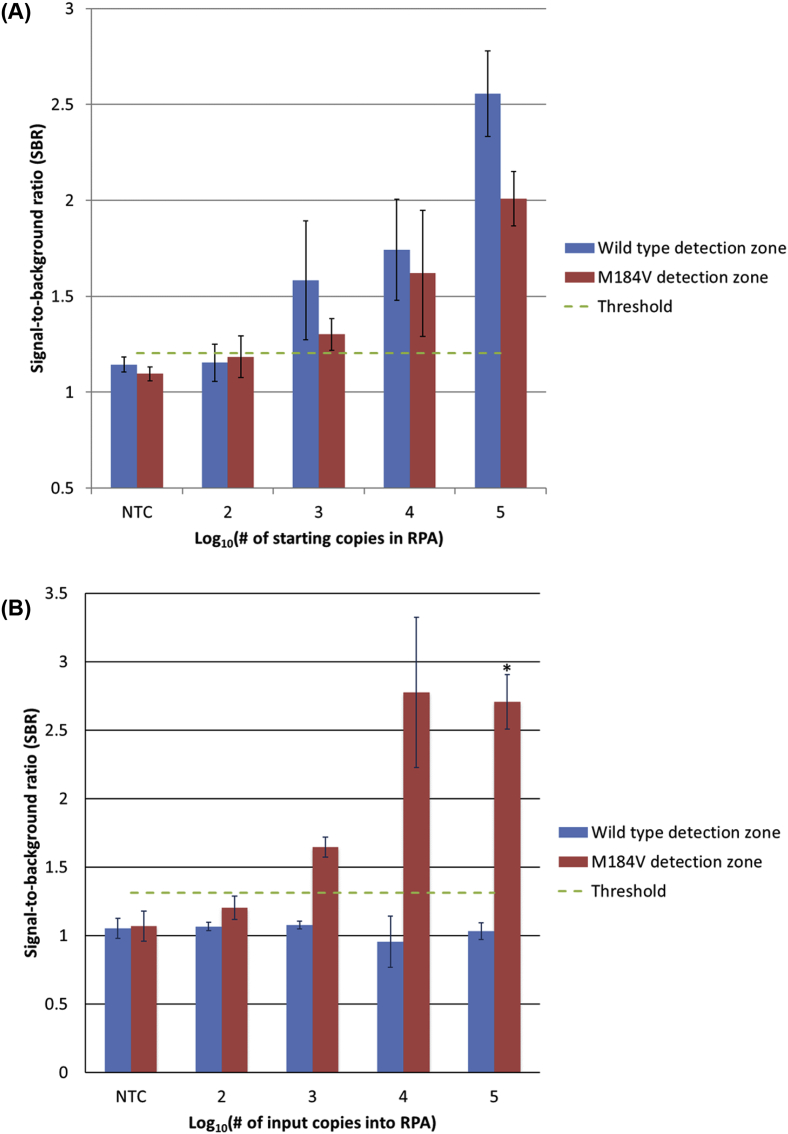
Results of the lateral flow assay for three separate experiments using wild type or M184V DNA. The threshold for a positive signal is defined as a signal-to-background ratio (SBR) greater than three standard deviations above the average SBR of all no-target samples. **(A)** Average SBRs of the lateral flow ELISA when varying copy numbers of wild type DNA are used as input to the RPA reaction (n = 3). Error bars show one standard deviation. **(B)** Average SBRs of the lateral flow ELISA when varying copy numbers of DNA containing the M184V mutation are used as input to the RPA reaction (n = 3, *n = 2). Error bars show one standard deviation.

**Fig. 7:**
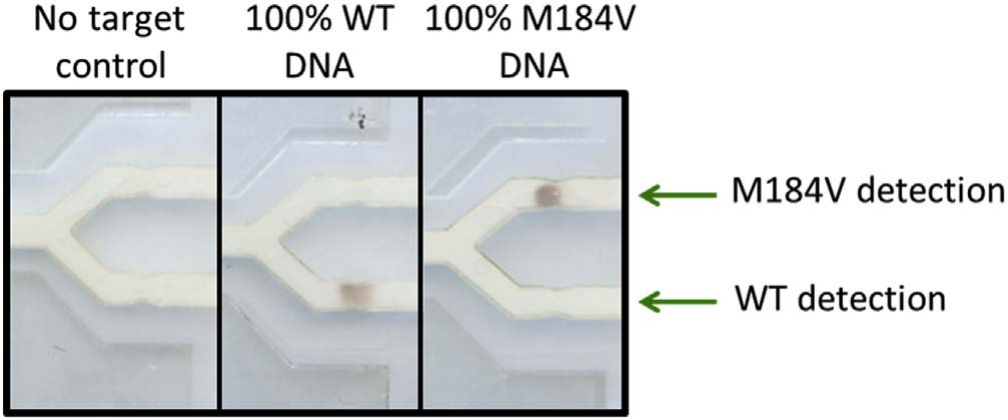
Specificity of the lateral flow assay. Scanned image of the detection regions of a representative set of lateral flow strips (10^5^ pre-amplification copies of DNA).

When the OLA product is transferred to the lateral flow assay, results show no nonspecific binding with wild type only and mutant only samples, down to a limit of detection of 1000 pre-amplification copies of DNA for wild type samples (Fig. 6A) and mutant samples (Fig. 6B). A representative set of lateral flow strips is shown in Fig. 7.

### Lateral flow assay limit of detection with a mixture of WT and mutant sequences

**Fig. 8:**
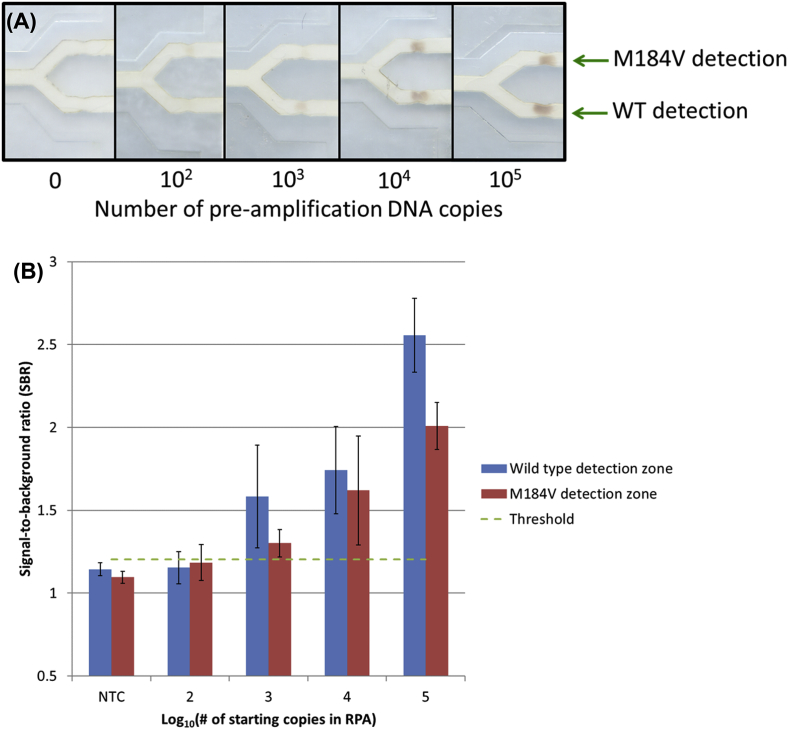
Products of OLA detected in the lateral flow ELISA format showing a limit of detection of 1000 total pre-amplification copies. **(A)** Image of a representative set of lateral flow strips containing varying pre-amplification copy numbers of 80% WT and 20% mutant DNA. **(B)** Average SBRs (n = 3) with a threshold for positivity of 1.203.

When tested with a mixture of 80% WT DNA and 20% mutant DNA, a threshold that corresponds to standard HIV drug resistance genotyping sensitivity by PCR and Sanger sequencing [27], the lateral flow assay was found to have a limit of detection of 10^3^ total pre-amplification copies (800 WT and 200 mutant DNA copies). A representative set of strips is shown in Fig. 8A. Fig. 8B shows that the signal-to-background ratios of each detection spot are proportional to the amount of input DNA. A positive signal is defined as having a SBR greater than three standard deviations above the average SBR of all no-target samples.

## Discussion

This paper describes two key steps that each make drug resistance detection more accessible: (1) isothermal amplification of a region that contains the major mutations that account for resistance to first-line therapies, and (2) lateral flow-based identification of M184V. The assay detects the presence of the most common HIV-1 drug resistance mutation, M184V, in a paper format following isothermal amplification by RPA. The device can distinguish between wild type and mutant DNA with high specificity, and currently has a limit of detection of 1000 copies of HIV-1 DNA in the pre-amplified sample at a mutant fraction of 20%. Amplified DNA is used in an OLA, and the detection of OLA products is accomplished in a paper LFA that uses inexpensive and disposable materials. The LFA requires only a few minutes of hands-on time in adding each ELISA reagent to a glass fiber pad, after which flow is powered by capillary action, and results are visible in 45 min. The assay currently costs $12.70 (Table S-2), less than 10% of the current cost of genotyping HIV *pol* [28].

Although an OLA can detect only one mutation at a time, the fragment of HIV *pol* amplified in this work contains several other high-profile mutations including K103N, Y181C, and V106M. Cross sectional data suggests that the four mutations M184V, K103N, Y181C, and V106M would identify at least 90% of patients with resistance on a first-line regimen [29]. Thus, detecting these drug resistance mutations would allow immediate management for the majority of patients, which would reduce the failure time, onward transmission of HIV, and the costs of follow-up visits.

This paper builds on previous work that has reported on PCR in combination with OLA (PCR-OLA) [18,22]. The assay reported here does not require a plate reader typically used to perform ELISAs; instead, reagents are applied to glass fiber pads and ELISA is performed in a paper and plastic device. While other work has reported paper-based detection of resistance mutations [22], this assay does not require manual user steps to transfer the paper to reagents; instead, the required reagents are wicked across the paper membrane using lateral flow principles. Thus, this paper represents an important step towards a POC test for HIV drug resistance.

A number of improvements are required to meet clinical performance needs. First, reverse transcriptase should be incorporated into amplification as RNA viral load is better correlated with therapy response, and the assay should be adapted to detect more than one major mutation simultaneously. Since the RPA primers used do not overlap any of the four major mutations, the same primers used for these experiments should theoretically be sufficient for a final integrated assay that detects multiple mutations. In order to detect additional mutations using OLA, different ligation probes would be required [16] along with additional branches of the lateral flow ELISA.

To reduce user steps and cost, the workflow should be automated and the purification steps following amplification and ligation should be eliminated. A recently developed device, the Multiplexable Autonomous Disposable Nucleic Acid Amplification Test (MAD NAAT) contains separate regions for sample preparation, amplification, and detection, illustrating the potential for a fully integrated device. Using multiple modules, a similar strategy could be employed for the HIV device described here [30]. The purification steps are designed to remove primers, nucleotides, enzymes, salts, and other impurities from the amplified or ligated sample. One way to eliminate purification following the OLA may be to use oligo probes bound in paper that are complementary to the existing probes to capture excess oligo probe prior to the detection spot. The exclusion of either purification step would also decrease the total cost of the assay by $2 (Table S-2). Since the fixed costs of RPA and the purification steps are the most expensive parts of the assay, eliminating the purification steps would also have the most significant impact on assay cost. Alternatively, an on-chip version of both purification steps could potentially be incorporated into the paper network using a high-salt binding buffer, wash step, and low-salt elution buffer.

To reduce the thermal requirements of this assay, the OLA should also be optimized so the use of a battery-powered or exothermic heater might be feasible. Ideally, sample preparation should be incorporated in the assay, and the possibility of performing both amplification and ligation in a single, isothermal step should be explored. Several groups are currently working on POC sample preparation for nucleic acid amplification tests [[30], [31], [32], [33], [34]]. Finally, based on previous work [34,35], the amplification step has the potential to be performed in paper.

## Conclusions

While much work remains to enable use of the integrated assay to detect HIV resistance mutations in clinical samples, this paper demonstrates proof-of-concept for a strategy that is feasible to use in a rural hospital laboratory. This assay represents a first step towards drug resistance screening at the POC and could be adapted for other infectious diseases. If implemented with a sample preparation method, this device could be part of a complete, sample-to-answer HIV-1 resistance assay that would make resistance testing more accessible to low-resource settings.

## Appendix A Supplementary data

Sequences of the forward and reverse primers used to develop the RPA assay and a cost of materials table indicating the cost of each reagent or material used in the assay (included in separate file)

## References

[1] UNAIDS (2016). Global AIDS Update 2016. http://www.unaids.org/sites/default/files/media_asset/global-AIDS-update-2016_en.pdf.

[2] UNAIDS (2017). Fact Sheet - Latest Global and Regional Statistics on the Status of the AIDS Epidemic. http://www.unaids.org/sites/default/files/media_asset/UNAIDS_FactSheet_en.pdf.

[3] World Health Organization (2016). Consolidated Guidelines on the Use of Antiretroviral Drugs for Treating and Preventing HIV Infection: Recommendations for a Public Health Approach. http://apps.who.int/iris/bitstream/10665/208825/1/9789241549684_eng.pdf?ua=1.

[4] UNAIDS (2014). 90-90-90 an Ambitious Treatment Target to Help End the AIDS Epidemic. http://www.unaids.org/Sites/Default/Files/Media_Asset/90-90-90_En_0.pdf.

[5] World Health Organization (2017). HIV Drug Resistance Report 2017. http://apps.who.int/iris/bitstream/10665/75183/1/9789241503938_eng.pdf.

[6] Barth R.E., van der Loeff M.F.S., Schuurman R., Hoepelman A.I., Wensing A.M. (2010). Virological Follow-up of Adult Patients in Antiretroviral Treatment Programmes in Sub-Saharan Africa: a Systematic Review.

[7] McMahon J.H., Elliott J.H., Bertagnolio S., Kubiak R., Jordan M.R. (2013). Viral suppression after 12 months of antiretroviral therapy in low- and middle-income countries: a systematic review. Bull. World Health Organ..

[8] Aghokeng A.F., Monleau M., Eymard-Duvernay S., Dagnra A., Kania D., Ngo-Giang-Huong N., Toni T.D., Touré-Kane C., Truong L.X.T., Delaporte E., Chaix M.L., Peeters M., Ayouba A. (2014). Extraordinary heterogeneity of virological outcomes in patients receiving highly antiretroviral therapy and monitored with the world health organization public health approach in sub-Saharan Africa and southeast Asia. Clin. Infect. Dis..

[9] Sigaloff K.C.E., Hamers R.L., Wallis C.L., Kityo C., Siwale M., Ive P., Botes M.E., Mandaliya K., Wellington M., Osibogun A., Stevens W.S., van Vugt M., Rinke de Wit T.F. (2011). Unnecessary antiretroviral treatment switches and accumulation of HIV resistance mutations; two arguments for viral load monitoring in Africa. J. Acquir. Immune Defic. Syndr..

[10] (2014). Joint United Nations Programme on HIV/AIDS (UNAIDS).

[11] Rhee S.-Y., Jordan M.R., Raizes E., Chua A., Parkin N., Kantor R., Van Zyl G.U., Mukui I., Hosseinipour M.C., Frenkel L.M., Ndembi N., Hamers R.L., Rinke de Wit T.F., Wallis C.L., Gupta R.K., Fokam J., Zeh C., Schapiro J.M., Carmona S., Katzenstein D., Tang M., Aghokeng A.F., De Oliveira T., Wensing A.M.J., Gallant J.E., Wainberg M.A., Richman D.D., Fitzgibbon J.E., Schito M., Bertagnolio S., Yang C., Shafer R.W. (2015). HIV-1 drug resistance mutations: potential applications for point-of-care genotypic resistance testing. PLoS One.

[12] Clutter D.S., Jordan M.R., Bertagnolio S., Shafer R.W. (2016). HIV-1 drug resistance and resistance testing. Infect. Genet. Evol..

[13] Daher R.K., Stewart G., Boissinot M., Bergeron M.G. (2016). Recombinase polymerase amplification for diagnostic applications. Clin. Chem..

[14] Crannell Z.A., Rohrman B., Richards-Kortum R. (2014). Quantification of HIV-1 DNA using real- time recombinase polymerase amplification. Anal. Chem..

[15] Rohrman B.A., Richards-Kortum R.R. (2012). A paper and plastic device for performing recombinase polymerase amplification of HIV DNA. Lab Chip.

[16] Boyle D.S., Lehman D.A., Lillis L. (2013). Rapid detection of HIV-1 proviral DNA for early infant diagnosis using rapid detection of HIV-1 proviral DNA for early infant diagnosis. MBio.

[17] Piepenburg O., Williams C.H., Stemple D.L., Armes N.A. (2006). DNA detection using recombination proteins. PLoS Biol..

[18] Bui M., Stone G.G., Almer L., Flamm R.K. (2003). PCR-oligonucleotide ligation assay for detection of point mutations associated with quinolone resistance in Streptococcus pneumoniae. Antimicrob. Agents Chemother..

[19] Edelstein R.E., Nickerson D.A., Tobe V.O., Manns-Arcuino L.A., Frenkel L.M. (1998). Oligonucleotide ligation assay for detecting mutations in the human immunodeficiency virus type 1 pol gene that are associated with resistance to zidovudine, didanosine, and lamivudine. J. Clin. Microbiol..

[20] Toubanaki D.K., Christopoulos T.K., Ioannou P.C., Flordellis C.S. (2009). Identification of single-nucleotide polymorphisms by the oligonucleotide ligation reaction: a DNA biosensor for simultaneous visual detection of both alleles. Anal. Chem..

[21] Beck I.A., Deng W., Payant R., Hall R., Bumgarner R.E., Mullins J.I., Frenkel L.M. (2014). Validation of an oligonucleotide ligation assay for quantification of human immunodeficiency virus type 1 drug-resistant mutants by use of massively parallel sequencing. J. Clin. Microbiol..

[22] Panpradist N., Beck I.A., Chung M.H., Kiarie J.N., Frenkel L.M., Lutz B.R. (2016). Simplified paper format for detecting HIV drug resistance in clinical specimens by oligonucleotide ligation. PLoS One.

[23] Min Xiao S., Qing Li G., Hua Li W., Qiong Zhou R., Wei Yang J. (2007). Development and application of nested PCR assay for detection of dairy cattle-derived cyclospora sp. Vet. Parasitol..

[24] Fu E., Liang T., Spicar-Mihalic P., Houghtaling J., Ramachandran S., Yager P. (2012). Two-dimensional paper network format that enables simple multistep assays for use in low-resource settings in the context of malaria antigen detection. Anal. Chem..

[25] Grant B.D., Smith C.A., Karvonen K., Richards-Kortum R. (2016). Highly sensitive two-dimensional paper network incorporating biotin-streptavidin for the detection of malaria. Anal. Chem..

[26] Ramachandran S., Fu E., Lutz B., Yager P. (2014). Long-term dry storage of an enzyme-based reagent system for ELISA in point-of-care devices. Analyst.

[27] Zagordi O., Klein R., Däumer M., Beerenwinkel N. (2010). Error correction of next-generation sequencing data and reliable estimation of HIV quasispecies. Nucleic Acids Res..

[28] Novitsky V., Zahralban-Steele M., McLane M.F., Moyo S., van Widenfelt E., Simani G., Joseph M., Essex M. (2015). Long-Range HIV Genotyping Using Viral RNA and Proviral DNA For analysis of HIV drug resistance and HIV clustering. J. Clin. Microbiol..

[29] Rhee S.Y., Blanco J.L., Jordan M.R., Taylor J., Lemey P., Varghese V., Hamers R.L., Bertagnolio S., de Wit T.F.R., Aghokeng A.F., Albert J., Avi R., Avila-Rios S., Bessong P.O., Brooks J.I., Boucher C.A.B., Brumme Z.L., Busch M.P., Bussmann H., Chaix M.L., Chin B.S., D'Aquin T.T., De Gascun C.F., Derache A., Descamps D., Deshpande A.K., Djoko C.F., Eshleman S.H., Fleury H., Frange P., Fujisaki S., Harrigan P.R., Hattori J., Holguin A., Hunt G.M., Ichimura H., Kaleebu P., Katzenstein D., Kiertiburanakul S., Kim J.H., Kim S.S., Li Y., Lutsar I., Morris L., Ndembi N., Paranjape K.P.N.G.R.S., Peeters M., Poljak M., Price M.A., Ragonnet-Cronin M.L., Reyes-Terán G., Rolland M., Sirivichayakul S., Smith D.M., Soares M.A., Soriano V.V., Ssemwanga D., Stanojevic M., Stefani M.A., Sugiura W., Sungkanuparph S., Tanuri A., Tee K.K., Truong H.H.M., van de Vijver D.A.M.C., Vidal N., Yang C., Yang R., Yebra G., Ioannidis J.P.A., Vandamme A.M., Shafer R.W., Geographic, Temporal (2015). Trends in the molecular epidemiology and genetic mechanisms of transmitted HIV-1 drug resistance: an individual-patient- and sequence-level meta-analysis. PLoS Med..

[30] Lafleur L.K., Bishop J.D., Heiniger E.K., Gallagher R.P., Wheeler M.D., Kauffman P., Zhang X., Kline E.C., Buser J.R., Kumar S., Byrnes S.A., Vermeulen N.M.J., Scarr N.K., Belousov Y., Mahoney W., Toley B.J., Ladd P.D., Lutz B.R., Yager P. (2014). A rapid, instrument-free, sample-to-result nucleic acid amplification test. Lab Chip.

[31] Tang R.H., Yang H., Choi J.R., Gong Y., Feng S.S., Pingguan-Murphy B., Huang Q.S., Shi J.L., Mei Q.B., Xu F. (2017). Advances in paper-based sample pretreatment for point-of-care testing. Crit. Rev. Biotechnol..

[32] Cui F., Rhee M., Singh A., Tripathi A. (2015). Microfluidic sample preparation for medical diagnostics. Annu. Rev. Biomed. Eng..

[33] Rodriguez N.M., Linnes J.C., Fan A., Ellenson C.K., Pollock N.R., Klapperich C.M. (2015). Paper-based RNA extraction, in situ isothermal amplification, and lateral flow detection for low-cost, rapid diagnosis of influenza a (H1N1) from clinical specimens. Anal. Chem..

[34] Rohrman B., Richards-Kortum R. (2015). Inhibition of Recombinase polymerase amplification by background DNA: a lateral flow-based method for enriching target DNA. Anal. Chem..

[35] LaBarre P., Hawkins K.R., Gerlach J., Wilmoth J., Beddoe A., Singleton J., Boyle D., Weigl B. (2011). A simple, inexpensive device for nucleic acid amplification without electricity-toward instrument-free molecular diagnostics in low-resource settings. PLoS One.

