# Supplementary material for: Paper-based detection of HIV-1 drug resistance using isothermal amplification and an oligonucleotide ligation assay
Source: Anal Biochem. 2018 Mar 1;544:64–71. doi: 10.1016/j.ab.2017.12.008 (PMC5854266; doi:10.1016/j.ab.2017.12.008)
Supplement: Supplementary material [file mmc1.docx]

Paper-based detection of HIV-1 drug resistance using isothermal amplification and an oligonucleotide ligation assay

Mary E. Natoli^a^, Brittany A. Rohrman^a^, Carolina de Santiago^a^, Gert U. van Zyl^b,c^, Rebecca R. Richards-Kortum^a^*

^a^Department of Bioengineering, Rice University, 6100 Main St MS-142, Houston, TX 77005, USA

^b^National Health Laboratory Service, Tygerberg Business Unit, Coastal Branch, South Africa

^c^Division of Medical Virology, Stellenbosch University, Parow, South Africa

*Corresponding author: [rkortum@rice.edu](mailto:rkortum@rice.edu), Address: Rice University Department of Bioengineering, 6100 Main St. MS-142, Houston, TX, 77030; Fax: 713-348-5877

This section contains the sequences of the forward and reverse primers used to develop the recombinase polymerase amplification (RPA) assay and a cost of materials table indicating the cost of each reagent or material used in the assay.

| **Candidate Forward Primers** | **Candidate Reverse Primers** |
| --- | --- |
| 4DR-F1: AATTAGGAATACCACATCCCGCAGGGTTAAAAAAG (2821🡪2855)  4DR-F2: AAGTTCAATTAGGAATACCACATCCCGCAGGG (2811🡪2846)  4DR-F3: CAATTAGGAATACCACATCCCGCAGGGTTAAA (2820🡪2851)  4DR-F4: CAATTAGGAATACCACATCCCGCAGGGTTAAAAAAG (2820🡪2855)  **4DR-F5: CTGGGAAGTTCAATTAGGAATACCACATCCCGC (2810🡪2842)**  4DR-F6: TTCAATTAGGAATACCACATCCCGCAGGG (2818🡪2846)  4DR-F7: AAGTTCAATTAGGAATACCACATCCCGCAGGG (2815🡪2846)  4DR-F8: TAGGAATACCACATCCCGCAGGGTTAAAAAAG (2824🡪2855) | 4DR-R1: CCTATTTCTAAGTCAGATCCTACATACAAATCATC (3101🡨3136)  4DR-R2: CCCTATT TCTAAGTCAGATCCTACATACAAATCATC (3101🡨3137)  4DR-R3: CTGCCCTATTTCTAAGTCAG ATCCTACATACAAATC (3105🡨3140)  4DR-R4: CTGCCCTATTTCTAAGTCAG ATCCTACATAC (3110🡨3140)  4DR-R5: CTATGCTGCCCTATTTCTAAGTCAGATCCTACATAC (3110🡨3145)  4DR-R6: CTATGCTGCCCTATTTCTAAGTCAGATCCTAC (3114🡨3145)  **4DR-R7: TTCTATGCTGCCCTATTTCTAAGTCAGATCCTAC (3114🡨3147)**  4DR-R8: TTGTTCTATGCTGCCCTATTTCTAAGTCAGATCC (3114 🡨3150) |

**Table S-1.** Candidate forward and reverse primers tested in the primer screen. This table lists the 420-bp sequence of the HIV genome targeted by the RPA assay and shows that the four major drug resistance mutations are contained within the sequence to be amplified. Candidate forward and reverse primers screened are listed. The primer pair chosen after the best amplification efficiency was 4DR-F5 and 4DR-R7 (**bolded**).

| **Material** | **Cost/rxn (USD)** |
| --- | --- |
| RPA kit | 3.781 |
| **OLA purification** | **2.156** |
| **RPA purification** | **2.136** |
| **SA-HRP40** | **2.000** |
| Taq ligase + buffer | 1.478 |
| Anti-FITC | 0.746 |
| Sticky acetate | 0.157 |
| Acetate | 0.076 |
| Anti-digoxigenin | 0.060 |
| Nitrocellulose paper | 0.023 |
| Oligo WT probe | 0.023 |
| Oligo mutant probe | 0.023 |
| Oligo common probe | 0.023 |
| Cellulose | 0.003 |
| PBST | 0.003 |
| Glass fiber | 0.001 |
| Diaminobenzidine | 0.001 |
| Sodium percarbonate | 0.000 |
|  |  |
| **Total assay cost** | **12.688** |

**Table S-2.** Cost of materials for the assay. Elements in bold are items that the authors believe could feasibly be reduced, which would bring the assay cost under $10.
